# Visual Suppression is Impaired in Spinocerebellar Ataxia Type 6 but Preserved in Benign Paroxysmal Positional Vertigo

**DOI:** 10.3390/diagnostics2040052

**Published:** 2012-10-11

**Authors:** Masahiko Kishi, Ryuji Sakakibara, Tomoe Yoshida, Masahiko Yamamoto, Mitsuya Suzuki, Manabu Kataoka, Yohei Tsuyusaki, Akihiko Tateno, Fuyuki Tateno

**Affiliations:** 1 Neurology, Internal Medicine, Sakura Medical Center, Toho University, Sakura, 564-1 Shimoshizu, Sakura 285-8741, Japan; E-Mails: neuro-mkishi@sakura.med.toho-u.ac.jp (M.K.); osirukooomori@yahoo.co.jp (Y.T.); tateno@med.toho-u.ac.jp (A.T.); f-tateno@sakura.med.toho-u.ac.jp (F.T.); 2 Department of Otolaryngology, Sakura Medical Center, Toho University, Sakura 285-0841, Japan; E-Mails: tomoe@med.toho-u.ac.jp (T.Y.); masa@med.toho-u.ac.jp (M.Y.); mitsuya.suzuki@med.toho-u.ac.jp (M.S.); 3 Clinical Physiology Unit, Sakura Medical Center, Toho University, Sakura 285-8741, Japan; E-Mail: kataoka@sakura.med.toho-u.ac.jp

**Keywords:** visual suppression test, spinocerebellar ataxia 6, benign paroxysmal positional vertigo, flocculus, nodulus

## Abstract

Positional vertigo is a common neurologic emergency and mostly the etiology is peripheral. However, central diseases may mimic peripheral positional vertigo at their initial presentation. We here describe the results of a visual suppression test in six patients with spinocerebellar ataxia type 6 (SCA6), a central positional vertigo, and nine patients with benign paroxysmal positional vertigo (BPPV), the major peripheral positional vertigo. As a result, the visual suppression value of both diseases differed significantly; e.g., 22.5% in SCA6 and 64.3% in BPPV (p < 0.001). There was a positive correlation between the visual suppression value and disease duration, cerebellar atrophy, and CAG repeat length of SCA6 but they were not statistically significant. In conclusion, the present study showed for the first time that visual suppression is impaired in SCA6, a central positional vertigo, but preserved in BPPV, the major peripheral positional vertigo, by directly comparing both groups. The abnormality in the SCA6 group presumably reflects dysfunction in the central visual fixation pathway at the cerebellar flocculus and nodulus. This simple test might aid differential diagnosis of peripheral and central positional vertigo at the earlier stage of disease.

## 1. Introduction

Early differential diagnosis of peripheral and central positional vertigo is still a challenge for neurologists. Positional vertigo is a common neurologic emergency and mostly the etiology is peripheral [1]. However, central diseases may mimic peripheral positional vertigo at their initial presentation [2,3,4,5,6,7,8]. We here describe the results of visual suppression test in six patients with spinocerebellar ataxia type 6 (SCA6), a central positional vertigo [2,3], and nine patients with benign paroxysmal positional vertigo (BPPV), the major peripheral positional vertigo [1]. 

## 2. Subjects and Methods

We had 15 individuals who complained of positional vertigo. Among these, six patients had genetically-diagnosed SCA6 (3 men, 3 women; mean age at onset 54.3 years (42–65 years); mean age at observation 64.3 years (56–75 years); mean disease duration 11 years (6–15 years; mean CAG repeat number 24.3 (21–26, normal < 13)) [2]. Neuro-otological examination showed cerebellar-type extra-ocular movement abnormality in cases 1, 2, 4, 5. These four cases had mild to moderate cerebellar ataxia in speech, limbs, and gait. In contrast, neuro-otological examination showed almost normal findings in cases 3 and 6 (Table 1). In both cases gait abnormality was only mild, and positional vertigo was still the major complaint. Nine age and sex-matched patients had BPPV [1], and these nine cases showed normal eye movement. All BPPV patients are thought to be idiopathic, and the diagnosis was based on the presence of positional nystagmus, which changes direction by changing the position of the head in the Dix-Hallpike maneuver.

Visual suppression (VS) test [9] was performed as follows. Under an electronystagmographic measurement, Caloric nystagmus was elicited by 8-degree-Celsius cold air flow introduced to the external auditory canal with the subjects’ eyes covered passively. Caloric nystagmus could be elicited in all subjects of SCA6 group and BPPV group. The room lights were then turned off. During this time the slow-phase velocity of the caloric nystagmus reached a maximum. The room lights were again turned on for 10 s with the subjects’ eyes open and fixed on a target. At the end of this period, the room lights were turned off again until the caloric nystagmus disappeared. We measured mean slow-phase velocity in dark (a) and in light (b), and calculated the VS value (%) as follows: (a − b)/a × 100. Statistics was analyzed using Spearman’s rank correlation. This study was approved by the local Ethics Committee. All individuals gave informed consent prior to participating in the study. 

**Table 1:** Extra-ocular movement examination in patients with spinocerebellar ataxia type 6 (SCA6) and paroxysmal positional vertigo (BPPV) (control).

	gaze-evoked nystagmus					eye-tracking test	optokinetic nystagmus	optokinetic post-nystagmus
	light room, eyes open			dark, eyes open	eyes closed			
	mid position	right 30 degree	left 30 degree	mid position	mid position			
case 1	downward	downward	downward	(−)	(−)	saccadic	normal	normal
case 2	downward, left	downward	downward	downward, left	to left	saccadic	abnormal	abnormal
case 3	(−)	(−)	(−)	(−)	to left	normal	normal	normal
case 4	downward, left	to right	to left	(−)	to left	saccadic	mildly abnormal	mildly abnormal
case 5	downward	(-)	to left	(−)	(−)	saccadic, rebound nystagmus	abnormal	abnormal
case 6	(−)	(−)	(−)	to left	(−)	not performed	abnormal	abnormal
control1	(−)	(−)	(−)	(−)	(−)	normal	normal	normal
control2	(−)	(−)	(−)	(−)	(−)	normal	normal	normal
control3	(−)	(−)	(−)	(−)	(−)	normal	normal	normal
control4	(−)	(−)	(−)	(−)	(−)	normal	normal	normal
control5	(−)	(−)	(−)	(−)	(−)	normal	normal	normal
control6	(−)	(−)	(−)	(−)	(−)	normal	normal	normal
control7	(−)	(−)	(−)	(−)	(−)	normal	normal	normal
control8	(−)	(−)	(−)	(−)	(−)	normal	normal	normal
control9	(−)	(−)	(−)	(−)	(−)	normal	normal	normal

## 3. Results

**Figure 1:**
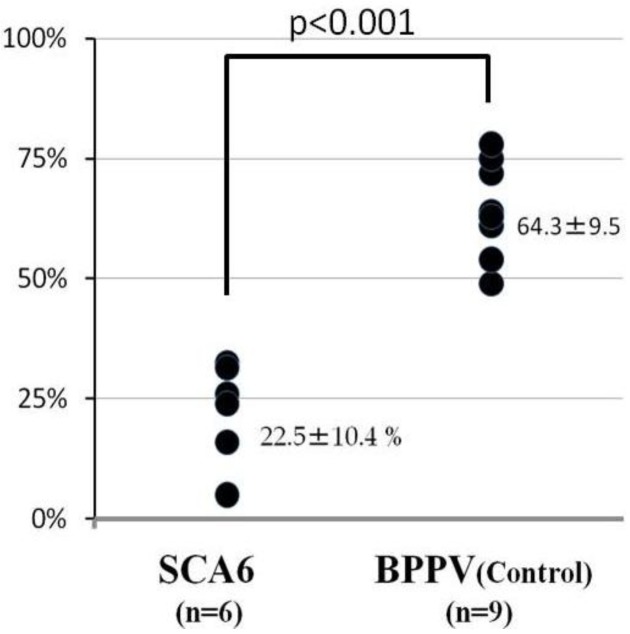
Results of the visual suppression test in SCA6 patients and control subjects. From a dark environment with the subjects’ eyes covered to a light environment with the subjects’ eyes open and fixed on a target, the slow-phase velocity of caloric nystagmus was suppressed in control subjects (64.3%), whereas the suppression was markedly reduced in SCA6 patients (22.5%) (p < 0.001). SCA6: spinocerebellar ataxia; BPPV: benign paroxysmal positional vertigo served as control.

The visual suppression (VS) value of both diseases differed significantly; e.g., 22.5% in SCA6 and 64.3% in BPPV, respectively (p < 0.001) (Figure 1). There was a positive correlation between the VS value and disease duration, cerebellar atrophy by a brain magnetic resonance imaging (MRI) scan, and CAG repeat length of SCA6 but they were not statistically significant. There was no clear correlation between VS value and eye tracking test abnormalities.

## 4. Discussion

Differential diagnosis of positional vertigo is still a challenge for neurologists. It can appear as a neurologic emergency and mostly the etiology is peripheral [1]. However, central diseases may mimic peripheral positional vertigo at their initial presentation, e.g., cerebellar (SCA6 [2,3], multiple system atrophy [4], paraneoplastic cerebellar degeneration [5], stroke [6]) as well as brainstem (stroke [7,8], *etc*.) pathologies. It is particularly important to provide proper management in these vertiginous subjects. Previously, Takahashi *et al*. [2] reported reduced VS value in 9 of 12 SCA6 subjects. Tsutsumi *et al*. [3] reported reduced VS value in 2 of 2 SCA6 subjects. The present study showed, for the first time to our knowledge, that visual suppression is impaired in SCA6, a central positional vertigo, but preserved in BPPV, the major peripheral positional vertigo, by directly comparing both groups. It is of importance that two of SCA6 group had only mild gait abnormality and almost normal eye movement, and positional vertigo was still the major complaint [10]. In both cases the VS value was abnormal. Therefore, in such cases, this simple test might aid differential diagnosis of peripheral and central positional vertigo at the earlier stage of disease.

Experimentally, Takemori *et al*. [11] studied VS following discrete lesions of various structures in the cerebellum of rhesus monkeys. They found that VS of Caloric nystagmus was lost completely to the ipsilateral side of the flocculus lesions. Nodulus lesions also resulted in a loss of VS, and this loss tended to recover in time. Extirpation or lesions of the uvula, vermis, para-flocculus, cerebellar cortex, or the fastigial or interpositus nuclei had no observed effect on the VS. They concluded that the flocculus and nodulus function as intermediators through which the visual system can modify or alter vestibular reflexes. We still did not know the detailed link between positional vertigo, reduced VS, and SCA6 pathology. However, SCA6 preferentially affects the cerebellum (including the vermis, flocculus and nodulus) and the inferior olive nucleus [2]. In light of Takemori’s experimental findings [11], atrophy in the cerebellar flocculus and nodulus might be the anatomical substrates for the reduced VS value in SCA6. 

In conclusion, the present study showed for the first time that visual suppression is impaired in SCA6, a central positional vertigo, but preserved in BPPV, the major peripheral positional vertigo, by directly comparing both groups. The abnormality in the SCA6 group presumably reflects dysfunction in the central visual fixation pathway at the cerebellar flocculus and nodulus. This simple test might aid differential diagnosis of peripheral and central positional vertigo at the earlier stage of disease.
